# Recent Advances in Nondestructive Method and Assessment of Corrosion Undercoating in Carbon–Steel Pipelines

**DOI:** 10.3390/s22176654

**Published:** 2022-09-02

**Authors:** Zazilah May, Md Khorshed Alam, Nazrul Anuar Nayan

**Affiliations:** 1 Electrical and Electronic Engineering Department, Universiti Teknologi PETRONAS, Seri Iskandar 32610, Perak, Malaysia; 2 Department of Electrical, Electronic and Systems Engineering, Faculty of Engineering and Built Environment, Universiti Kebangsaan Malaysia, Bangi 43600, Selangor, Malaysia

**Keywords:** acoustic emission, corrosion prediction, nondestructive testing, oil and gas industry, pipeline corrosion, structural health monitoring

## Abstract

Carbon–steel pipelines have mostly been utilized in the oil and gas (OG) industry owing to their strength and cost-effectiveness. However, the detection of corrosion under coating poses challenges for nondestructive (ND) pipeline monitoring techniques. One of the challenges is inaccessibility because of the pipeline structure, which leads to undetected corrosion, which possibly leads to catastrophic failure. The drawbacks of the existing ND methods for corrosion monitoring increase the need for novel frameworks in feature extraction, detection, and characterization of corrosion. This study begins with the explanations of the various types of corrosion in the carbon–steel pipeline in the OG industry and its prevention methods. A review of critical sensors integrated with various current ND corrosion monitoring systems is then presented. The importance of acoustic emission (AE) techniques over other ND methods is explained. AE data preprocessing methods are discussed. Several AE-based corrosion detection, prediction, and reliability assessment models for online pipeline condition monitoring are then highlighted. Finally, a discussion with future perspectives on corrosion monitoring followed by the significance and advantages of the emerging AE-based ND monitoring techniques is presented. The trends and identified issues are summarized with several recommendations for improvement in the OG industry.

## 1. Introduction

**Figure 1:**
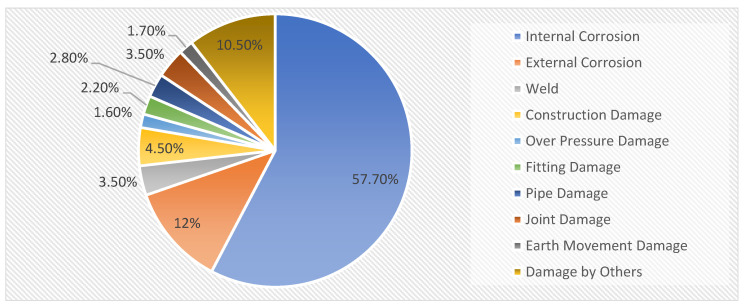
The percentages of several causes of production pipeline failure based on the report in [5].

Corrosion assessment and monitoring are valuable for applications in the oil and gas (OG) industry because corrosion factors lead to great economic damage, which is a large amount of the total global cost for OG industries yearly [1,2,3]. The impact of corrosion in the field of OG industries can be assessed based on its effect on operational expenditure and capital expenditure, including health, safety, and environment [4]. According to the production pipeline failure data between 1980 and 2005 in Alberta [5], Canada, corrosion is one of the main factors causing a pipeline failure. Figure 1 shows the percentages of several causes that contribute to pipeline failure.

Based on corrosion management statistics, the global cost caused by corrosion is billions of dollars annually [6]. Thus, the OG industries focus on the development of corrosion management strategies and control practices to significantly reduce the corrosion cost. Understanding the causes of corrosion mainly helps to implement and upgrade corrosion management and prevention strategies [7].

**Figure 2:**
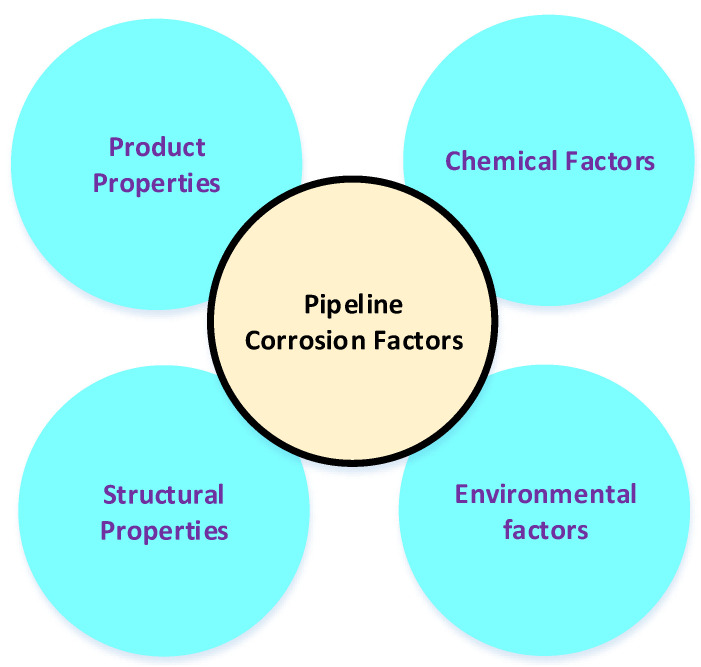
The causes of pipeline corrosion.

Corrosion of the carbon–steel pipeline can be attributed to several causes, including structural properties, materials properties, chemical factors, and environmental factors, as shown in Figure 2. These factors can further breakdown individually for better understanding. For example, material properties, residual and operating stresses, design factors, crevices, deposits, and others are associated with structural properties; temperature, pressure, flow rate, entrained solid and liquid and flow pattern are related to product properties; chemical factors included $H_{2}O$, $H_{2}S$, $CO_{2}$, dissolved solids, organic and inorganic acids, sulphur and sulphur compounds, microorganisms, hydrocarbons and pH; environmental factors associated with oil field composition, soil composition, temperature and moisture levels, saltwater, freshwater and land [8]. This article focuses on numerous methods developed for corrosion assessment and monitoring in OG industries.

The nondestructive (ND) technique represents the execution of material fault identification, which prevents the risk of material failure and avoids the interruption of future performance of the material. The degradation of the materials’ properties is known as corrosion, which is caused by environmental interactions [9]. The corrodible materials include metals and alloys, carbon steel, nonmetals, ceramics, and composites [10]. Owing to its economy and mechanical strength, mild steel is yet preferred for many applications. The fact that mild steel corrodes easily is the biggest disadvantage as it quickly loses strength, resulting in the failure of the structure. Carbon–steel or other metal structures, in particular, vessels, are routinely coated to avoid corrosion. The main goal of coating is to establish a barrier between the metal surface and the environment, avoiding corrosion. Although the coating protects the metal from corrosion, it is still susceptible to corrosion. The corrosion may happen on the coated carbon–steel structure, which is difficult to identify as the corrosion is hidden by the coating layer. Corrosion that goes unnoticed can lead to catastrophic failure. The risk to people’s safety, environmental degradation, and economic ramifications are all serious effects. When carbon steel comes into touch with water and oxygen, it corrodes. To boost the efficiency of inspection, many ND techniques have been used to identify corrosion beneath coating without removing it [11,12,13], Each of them has distinct capabilities. These ND methods are then used to identify problem areas that require more examination.

This study investigates the advancements in sensing technologies for corrosion data acquisition, corrosion data analysis methods, pipeline monitoring technologies, machine learning (ML) models for corrosion assessment, and reliability prediction. The objectives are to enhance the reliability of the coated carbon–steel pipeline and reduce the cost associated with corrosion. This paper is organized as follows. Section 2 discusses the existing several kinds of corrosion and its issues with control practices. Section 3 highlights the sensors that are utilized for corrosion data extraction. Section 4 explains several corrosion monitoring technologies utilized for carbon–steel pipelines in the OG industry. Section 5 explains the importance of AE methods over other ND methods. Section 6 studies the methods for corrosion data analysis, detection, and classification. Section 7 describes several corrosion assessment and reliability prediction models for the carbon–steel pipeline undercoating. Section 8 highlights the challenges and recommendations for future perspectives. Finally, Section 9 concludes this study.

## 2. Corrosion in Carbon–Steel Pipeline and Its Factors with Prevention Methods

Several kinds of corrosion and the causes that introduce corrosion in carbon–steel pipelines are stated and explained in the following subsections [14,15]. Various preventive methods are introduced to control corrosion and to enhance the sustainability of the pipelines [16]. Table 1 presents different types of corrosion and their factors with the preventive technique, which are explained in the next sub-sections.

### 2.1. Corrosion Types in Carbon–Steel Pipeline

#### 2.1.1. Uniform Corrosion

The most prevalent type of pipeline corrosion is uniform corrosion, which spreads consistently over exposed surfaces [14]. Uniform corrosion is a regular occurrence in the OG business and results in a consistent pipe wall reduction [17]. In the presence of corrosive species that can rapidly deteriorate metallic surfaces owing to either anodic or cathodic reactivity, untreated surfaces are susceptible to uniform corrosion. The most prevalent source of uniform corrosion, which is easily detected by rust, is oxidation in the presence of air or water. Corrosion inhibitors, protective coatings, and cathodic protection are all common strategies for preventing uniform corrosion [18].

#### 2.1.2. Pitting Corrosion

Pitting corrosion happens along treated surfaces where the base material has abrasions or inclusions, or when the surface is not appropriately treated to withstand environmental effects or is exposed to high concentrations of aggressive chemical species [14]. Pitting corrosion can be caused by a variety of factors [19]. Moreover, when the temperature is above the critical pitting temperature (CPT) defined by the American Society for Testing and Materials (ASTM) Standard G48-03, the resistance of a material to pitting corrosion is greatly diminished. Proper material selection, pH management, and cathodic or anodic protection are used to prevent pitting corrosion [20].

#### 2.1.3. Crevice Corrosion

Crevice corrosion is a type of corrosion usually introduced in a small area at or near a joint [14]. An electrochemical concentration cell is formed by the gap or fissure between the attached materials. Chlorides condense in the crevice, which lose oxygen and have a lower pH, making them an anode in an electrochemical reaction. Crevice corrosion is highly influenced by crevice geometry, material composition, and environmental conditions [21]. The critical crevice temperature (CCT) is defined by ASTM Standard G48-03 as the lowest temperature known to induce crevice corrosion. CCT is often substantially lower than CPT. Crevice corrosion can be reduced by using welded butt joints, solid non-absorbent gaskets, and metals with higher resistance to crevice corrosion. Continuous welding or soldering should be used to eliminate crevices in lap joints.

### 2.2. The Causes That Introduce Corrosion in Carbon–Steel Pipeline

#### 2.2.1. Galvanic Corrosion

When two metallic or semi-metallic conductors with different electrochemical potentials are exposed to an electrolytic fluid, galvanic corrosion, also known as bimetallic corrosion, occurs [14]. The electrochemical potential is dependent on the electrolyte and ambient circumstances. The main driving force to introduce galvanic corrosion is the variation in potential as the direction of current flows through the electrolyte to the direction of more noble metal, whereas galvanic corrosion occurs on the less noble metal or active metal [22]. Threaded junctions between materials that are wide apart in the galvanic series should be avoided. Moreover, dissimilar metals must be insulated whenever possible, and coatings should be applied and maintained for integrity to prevent galvanic corrosion.

#### 2.2.2. Cavitation and Erosive Corrosion

Cavitation corrosion is an erosion process that occurs when gas bubbles “implode” on a metal surface [23]. This corrosion is commonly connected to pressure variations brought on by the fluid’s hydrodynamic properties (e.g., propellers, stirrer blades, and hydraulic turbine blades). The value of a constant hydraulic regime in the fluid is impossible to be overestimated. When the surface is in good condition, the creation of vapor bubbles reduces in the potential sites. The increment of fluid pressure is typically adequate to provide a single phase-based fluid and avoid vapor bubble formation. Although adhesion concerns between the coating and the metal are a regular stumbling point, plastic or rubber coatings have consistently been shown to be useful. Erosive corrosion is typically associated with the areas, where varying fluid flows, increasing turbulence, the corrosiveness of the fluid, and some other factors [14]. The variation of erosion-corrosion rates is related to the pipeline material properties, fluid properties, flow regime, fluid velocities, particle sizes, geometry, and densities. The impacts of solid particles, such as sand, and their characteristics, such as geometry, sharpness, and hardness, are studied. Pollutants and corrosive species, such as oxygen and water, including salts, in particular, bicarbonate, chloride, and sulfate, accelerate erosion corrosion [24].

#### 2.2.3. Stray Current Corrosion

Stray current corrosion refers to the passing of electrical current through circuits other than the dedicated paths. The stray current corrosion occurs in the pipeline, whereas the flow of current travels from one location to another into the pipeline and then returns to the power source. Considering the process of the current flow, stray current corrosion introduced in the pipeline is proportional to the amount of current flowing during the process in the pipeline. Stray current corrosion can pose a threat to buried carbon–steel pipelines and other underground metallic infrastructure [25]. However, preventing them from the stray current corrosion is difficult because it is influenced by a variety of circumstances. Stray current corrosion on buried carbon–steel pipelines can be prevented using two basic methods, namely, the use of coating method and the use of cathodic protection method [26]. In the real environment, keeping the carbon–steel pipeline away from the direct or indirect connection between the power transmission system and the available stray current in the pipeline is crucial. Therefore, the purpose of general protection is to keep the potential of underground carbon–steel pipelines constant.

#### 2.2.4. Stress Corrosion Cracking (SCC)

Pipeline environmentally assisted cracking (EAC) is a kind of SCC. SCC is described as the formation of cracks in a pipeline as a consequence of a mix of factors, including the surrounding environment [14]. The pipe’s pressure carrying capacity is diminished when these determinants are combined. When water (electrolyte) comes into touch with steel, the minerals, ions, and gases in the water corrode the steel. Chemical or electrochemical reactions can cause general thinning, corrosion pits, and/or fractures [27].

In EAC, corrosion fatigue and SCC are two phenomena to be aware of. Corrosion fatigue is caused by chemically reactive substances penetrating fatigue fractures. These chemicals can speed up fracture propagation. Within the crack, the chemical environment might be more hostile than on the free surface [28]. Even if the metal at the fracture tip passives (forms an inert barrier), fatigue loading can break the brittle deposit, reactivating the process. Corrosion fatigue occurs when cyclic stress and a corrosive environment combine to lower the number of cycles required to break down. The corrosive environment’s major role, compared with the life of the pipe when no corrosion is present, is to shorten the component’s life [29]. SCC possesses corrosive processes and is sensitive to an aggressive environment and tensile stress. Tensile tension, which can be applied directly or as residual stress, produces fractures in the material. SCC is caused by long-term tensile stresses, whereas corrosion fatigue is caused by cyclic loading.

#### 2.2.5. Microbiologically Influenced Corrosion (MIC)

MIC occurs when either aerobic or anaerobic microorganisms cause corrosion to accelerate. Sulfate-reducing bacteria is a type of anaerobic bacterium that causes the majority of accelerated corrosion in offshore steel structures and marine environments [30]. On pipes that are predisposed to elevated levels of sulfides, regular pigging or cleaning operations should be performed to prevent MIC [31]. Chemical treatments or inhibitors, such as biocides, can be utilized to control the number of bacteria in a fluid.

## 3. Critical Sensors for Corrosion Monitoring in Carbon–Steel Pipeline

**Figure 3:**
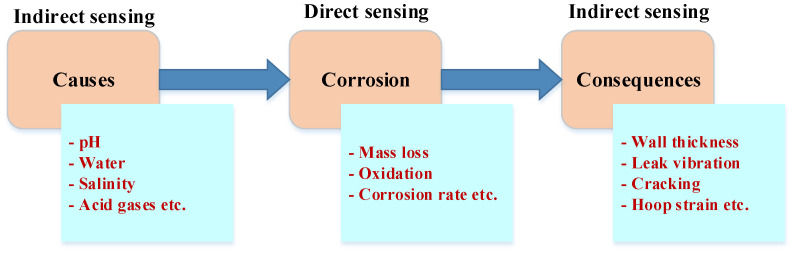
The ways of corrosion sensing to assess corrosion process from causes to consequences [33].

A range of corrosion sensor technologies based on distinct sensing ideas has been developed for various forms of corrosion. Two different types of corrosion sensors exist, that are, direct and indirect corrosion sensors [32], as shown in Figure 3. The detection of corrosion processes and corrosion rates introduced owing to a variety of corrosion causes and corrosive conditions are monitored directly using the direct corrosion sensors. Corrosion causes, such as water, low pH, and $CO_{2}$, or effects, such as rust, are monitored using indirect corrosion sensors. Corrosion processes and effects can be understood through the selection of monitoring parameters or areas of interest for sensing. Table 2 presents the existing corrosion sensors utilized widely and available commercially for corrosion monitoring in OG industries.

### 3.1. Corrosion Coupon

For OG infrastructures, corrosion coupons are utilized as point sensors with a small sensing coverage. Average corrosion rates are provided instead of real-time data over time by these types of sensors. The coupon sensors are placed for monitoring a corrosive environment over time and then extracting the measurement of corrosion weight [34]. The installation and removal of these sensors after corrosion lab examination, from another aspect, take a long time.

### 3.2. Electrical Resistance (ER) Probe

Point sensors, such as ER probes, may detect homogeneous corrosion in specific locations. As the number of sensing sites increases, the total cost also increases. Installation locations for ER probes, such as corrosion coupon sensors, must be carefully chosen to maximize their use. Placements are usually made based on experience, and even in the face of severe corrosion, some atypical placements can be ignored. The electrical-based sensor measurement of the ER probes enables the collection and log of electronic data [35]. However, ER probes may introduce some common electronic issues considering the collection of data logging, which is required to be maintained regularly and replaced. Electrical-based sensors for corrosion monitoring in carbon–steel pipelines must also conform to fundamental electrical safety rules.

### 3.3. Electrochemical Sensor

An electrochemical sensor makes use of intrinsic electrochemical methods, such as linear polarization resistance (LPR), galvanic current measurement, and electrochemical impedance spectroscopy to exploit the fundamental electrochemical features of corrosion [36]. Electrochemical sensors have several advantages, including direct detection [37]. Moreover, direct determination of electrochemical corrosion rates and the capability to examine in-situ corrosion techniques based on a range of electrochemical mechanisms are some more pros of electrochemical sensors. The electrochemical sensors are the most commercialized because of their simple operation and data interpretation in terms of the detection of pipeline corrosion using the LPR technique. The main disadvantages of these sensors are that externally forced potential or current might cause rapid corrosion compared with the true value. Hence, correct electrochemical parameter settings and electrode system design must be carefully specified. Furthermore, these sensors typically need an ion-conductive electrolyte, such as aqueous processes, and are not easily adaptable to non-conductive conditions without additional changes [38].

### 3.4. Ultrasonic Sensor

An ultrasonic sensor is placed to monitor pipeline corrosion and structural health by analyzing the wall thickness of the pipeline [39]. High-frequency (MHz) acoustic waves are generated from the piezoelectric transducer and controlled through electric pulses. These waves are produced perpendicular to the pipeline wall in an ultrasonic sensor. The generated acoustic waves are then reflected by the transducer by the external or inner surfaces. Portable and fixed ultrasonic corrosion sensors are available [40], and they can also be incorporated with in-line inspection systems. However, casing scales and highly attenuating muds can damage these acoustic-based sensors [41].

### 3.5. Multi-Frequency Electromagnetic Sensor

A multi-frequency electromagnetic sensor is widely utilized as a ND sensor for corrosion monitoring in the OG industry. Corrosion and pipeline integrity, for example, might be detected using a multi-frequency electromagnetic monitoring sensor [42]. The sensor is made of a transmitter coil and a receiver coil. An alternating current (AC) activates the transmitter coil, and the alternating magnetic field generates an eddy current in the conductive pipeline surrounding [43]. The initial electromagnetic field from the transmitter, in combination with a secondary electromagnetic field from eddy currents in the carbon–steel pipelines, causes a phase-shifted voltage in a distinct receiver coil. The phase shift and the change of magnitude are influenced by the electrical conductivity of the carbon–steel materials and the presence of defects. Owing to the skin effect, low-frequency electromagnetic scans can compute pipe metal thickness, whereas high-frequency electromagnetic scans can detect internal wall properties [44].

### 3.6. Optical Fiber Sensor (OFS)

The OFS is another ND sensor developed in recent years for corrosion monitoring [45] which is small in size, flexible, lightweight, and highly sensitive [46]. Moreover, the OFS is compatible with optical fiber data communication networks and has enhanced safety in the existence of flammables compared with other electrical-based sensors [47]. The traditional point sensor monitors pipeline corrosion at distinct areas that are evaluated individually by different channels, that is, each sensor detects just a single point. By contrast, OFS can measure corrosion in several distinct areas inside a single optical channel.

### 3.7. Passive Radio-Frequency Identification (RFID) Sensor

The RFID sensor, particularly the chipless passive RFID sensor, is a comprehensive family of wireless sensors for pipeline corrosion and SHM with cheap cost, compact size, lightweight, and remote sensing [48]. RFID sensors can detect pipeline corrosion directly when they are paired with corrosion-sensitive structures in the sensor design. The circuit on an RFID sensor for metal or steel will not be suitably electrified through the antennas to respond because of the corrosion of the connection between the circuit and the antennae, indicating the presence of corrosion [49]. Furthermore, RFID sensors may be utilized as an indirect method to monitor pipeline corrosion.

### 3.8. Acoustic Emission (AE) Sensor

The waves are recorded using AE sensors, which are non-intrusive and passive sensors affixed to the surface, and the signals are transferred to the AE acquisition system for further analysis [50]. Stress waves are only generated through active or increasing corrosion or cracks; if corrosion or cracks exist but remain constant of the corrosion growth, then no AE is received. The AE sensors are often coated with target-specific chemical-sensitive materials, such as polymers, metals, and metal oxides, and are tiny in size, low-cost, and efficient. Rayleigh waves are most typically used for gaseous phase sensing with gas absorbing or reactive layers deposited on AE devices as functional sensing layers [51].

## 4. Existing ND Techniques for Corrosion Monitoring

Several ND corrosion monitoring methods developed in OG industries have been reviewed and explained in the following subsections. Table 3 presents a summary of these methods.

### 4.1. Ultrasonic Monitoring Method

The ultrasonic method works by generating mechanical waves or vibrations in the specimens that are being tested in an experimental lab or real environment [52]. Solids are not the only type of sample available. Most ultrasonic techniques use 1 to 100 MHz as operating frequencies. The term “ultrasonic” describes sound frequencies that are above the range of human hearing. The density and elastic modulus of a material determine how fast ultrasonic waves travel through it. Hence, ultrasonic procedures are ideal for determining the characteristics of materials. Furthermore, changes in material characteristics at the borders will substantially reflect ultrasonic waves. As a result, ultrasonic technologies are frequently utilized for thickness measurement and corrosion monitoring [53].

**Figure 4:**
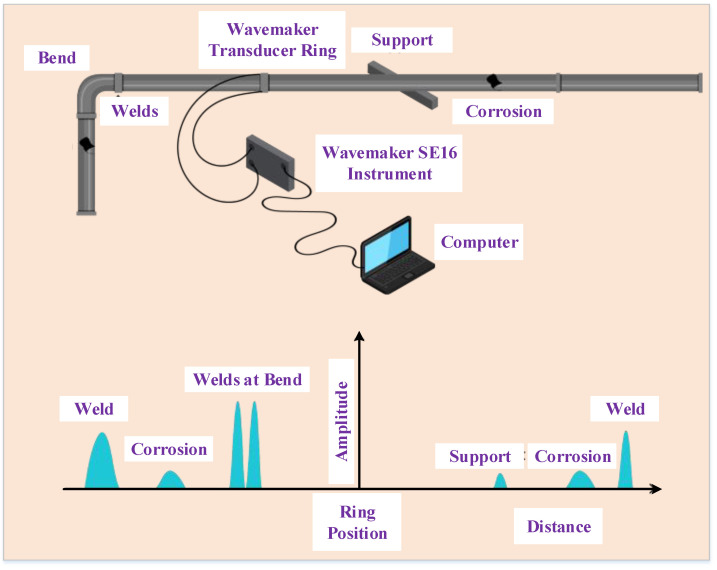
Ultrasonic monitoring for corrosion assessments adopted from [55].

Although ultrasonic methods can detect corrosion, distinguishing between reflections from the corroded surface or near corroded surface and reflections from the surfaces of the other material with these approaches is difficult. An additional issue is that acoustically linking pulses from the transducer to the material requires a coupling medium, such as water or gel. Owing to the surface preparation requirements, traditional ultrasonic methods are not suitable for some scenarios. In recent years, considerable effort has gone into developing non-contact ultrasonic techniques for defect assessment [54] that do not require surface preparation. Conventional piezoelectric transducers [54], which are more appropriate for most inspection situations, are used in air-coupled procedures. An adequate angle for introducing the ultrasound into the specimens to be inspected, from another aspect, is a strict condition. An experimental set for measuring corrosion in pipes using guided acoustic waves has been published, as shown in Figure 4. A ring transducer that runs through the pipe emits guided sound waves. When these sound waves hit corrosion on the walls of the pipelines, they are reflected and back to the transducer [55].

### 4.2. Laser Ultrasonic Method

**Figure 5:**
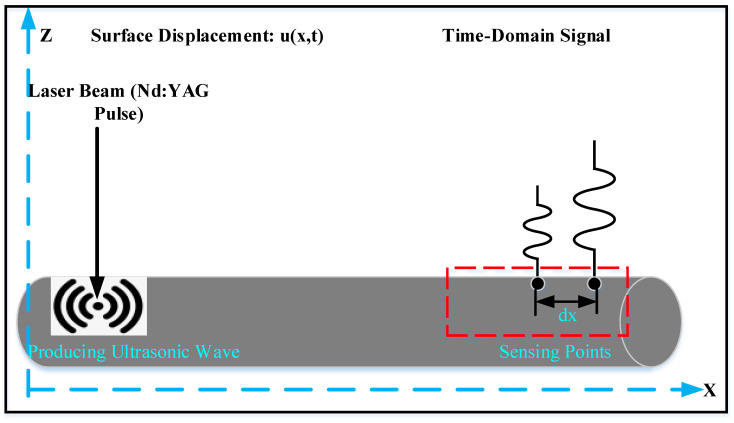
Schematic for laser ultrasonic corrosion monitoring method [58].

Owing to the recent advancements in laser ultrasonic technology, lasers may now be utilized to make and identify ultrasonic waves [56]. This ND technique has been utilized to characterize materials, quantify material thickness, and find faults. A laser ultrasonic generator produces a laser ultrasonic device including an interferometry sensor and transducer, which are all run through the pipelines. Internal corrosion in hollow metallic components was detected and located using the laser ultrasonic of Liu et al. [57]. Piezoelectric transducers with an operating frequency of 125 MHz and broadband laser-ultrasonic are utilized to create waves that interact with the pipeline corrosion. Then, the variation in the generated wave modes is assessed based on time-frequency decomposition methods. A laser beam is created and senses the non-corrosive surface at the face side, where the reflection method is employed, as illustrated in Figure 5. A pulsed Nd: YAG laser has been utilized to make an ultrasound [58]. A TEMPO laser ultrasonic receiver detects the reflected ultrasound. The detector generates an analog voltage over time, which is proportional to the instantaneous displacement at ultrasonic frequencies. Scanning the specimens to detect pipeline corrosion can generate geometric images of corrosion. The main drawback of this method is that it requires access to the material surface of the specimen under test. Moreover, laser-ultrasonic applications are very limited and sensitive to surface-breaking issues.

### 4.3. OFS Monitoring Method

The OFS is an emerging method to monitor carbon–steel corrosion in recent years [59,60,61]. When a light wave transmits in an optical fiber, the change in the electric field of the light produces acoustic waves over electrostriction, periodically altering the fiber refractive index. A pump pulse and a probe continuous wave are started from the two ends of an optical fiber and counter propagate along the fiber, which can be measured by the optimal time domain reflectometry. This method has numerous merits, such as high precision, sensitivity, and immunity to electromagnetic interference, compared with other traditional electric corrosion sensor-based methods.

**Figure 6:**
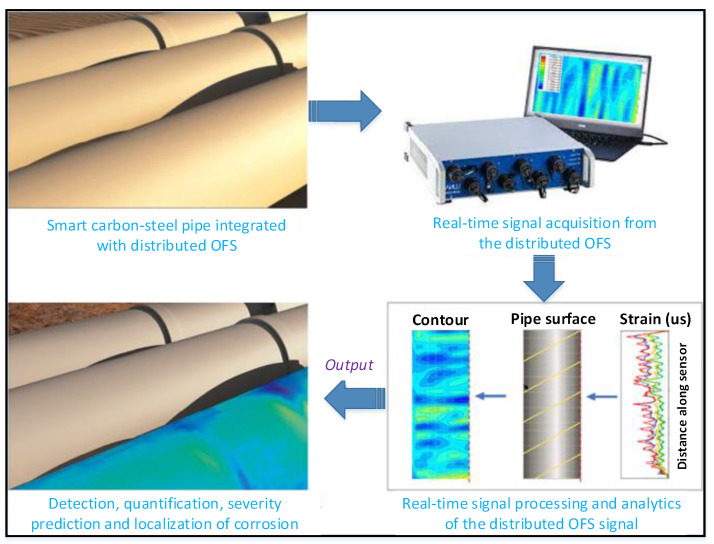
Schematic for distributed OFS based corrosion monitoring method [63].

The OFS method can be classified into two categories, namely, point sensor-based and distributed sensor-based methods, in terms of their sensing length. As the conditions of corrosion vary in a structure, a large number of point sensors are required for corrosion monitoring in a large-scale structure. From another aspect, the distributed sensors have a great potential compared with the point sensor in terms of providing detailed results and high spatial resolution. Distributed optical fiber sensing can continuously monitor the parameters along the whole optical fiber with a specific spatial resolution by interrogating the backscattered light continuously [62]. Figure 6 presents the distributed OFS based method for pipeline corrosion monitoring [63]. The corrosion growth of a pipe was detected through the variation of strains sensed by a distributed OFS. The distributed OFS is placed on the pipe with the location, and the length along the distributed OFS can be correlated with the position on the pipe’s surface. The strain distributions are computed from the distributed OFS, which can be replotted with the correlation over a coordinate transform. The distributed OFS position on the pipes is explained in a polar coordinate structure, which can also be converted into the Cartesian coordinate structure. The specimen is separated along the length of the pipe, and the circumference of the pipe is unfolded to a flat plane, as shown in Figure 6. The circumference becomes the plane width, and the pipe length remains the same length as the plane. Then, the measured strain distributions are mapped in the plane to present a two-dimensional contour of the strain, which can be utilized for visualization of the condition of corrosion in real time. The highlighted areas with the red color of pipes are referred to high strains, which indicate the appearance of severe corrosion. However, more analysis and investigation are needed for the evaluation of the effects of different parameters of this monitoring method.

### 4.4. Eddy Current Monitoring Method

**Figure 7:**
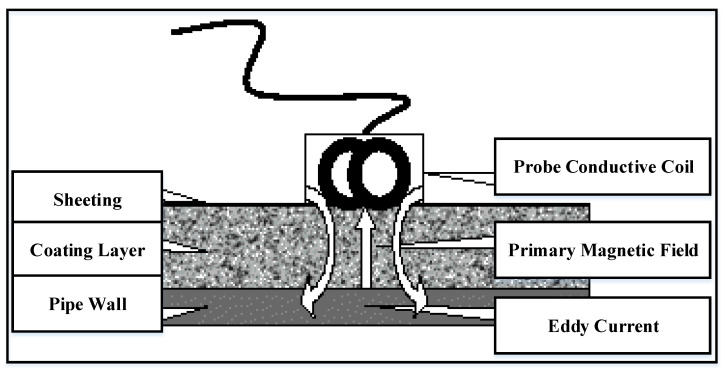
Eddy current framework for corrosion detection and monitoring [64].

One of the most useful methods is eddy current monitoring, which detects and assesses the corrosion of the conductive materials. In this technique, the conducting coil, including an AC, is placed near the specimen to form an initial magnetic field around the coil in an axial direction [64]. From another aspect, the electrical current forms its own secondary magnetic field in the opposite direction from the magnetic field of the conducting coil, which contradicts Lenz’s Law [65]. The interaction between the conducting coil’s magnetic field and eddy current magnetic field is studied using sensors or coils, as shown in Figure 7.

Eddy current approaches are used to identify the existence of different types of corrosion on the surface of carbon–steel pipelines and have proven to be quite successful. As a result, eddy current methods have become the most popular ND methods, as they are exceedingly portable and reasonably inexpensive. For SCC inspection, researchers introduced a novel eddy current array technique [66]. Traditional eddy current approaches, from another aspect, face difficulties in the detection and assessment of tiny metal losses caused by corrosion. The main reason is the skin effect having a significant impact on the effectiveness of eddy current approaches to detect subsurface problems. The majority of the current flow occurs on the conductor’s surface, with an exponential decline as depth increases because of this skin effect. A multifrequency eddy current and signal processing techniques have been introduced in [67] to characterize and monitor the iron oxide on the storage tank. The proposed techniques were used to map the lap joint thicknesses in different layers. The findings indicate that the corrosion estimation error is less than 5% for the thickness of various corrosion. Meanwhile, novel magnetic sensors have been created to replace coils in eddy current technology [68]. The authors in [69] investigated the utilization of a giant magnetoresistive (GMR) sensor array to evaluate different types of corrosion in pipelines that have been coated. Moreover, the implementation of GMR sensors has been thoroughly studied and explained by Rifai et al. [70]. Eddy current-based approaches, from another aspect, are confined to electrically conductive materials. Additionally, these technologies are extremely sensitive to lift-off effects, and they require access to the surface of the materials.

### 4.5. Magnetic Flux Leakage (MFL) Monitoring Method

**Figure 8:**
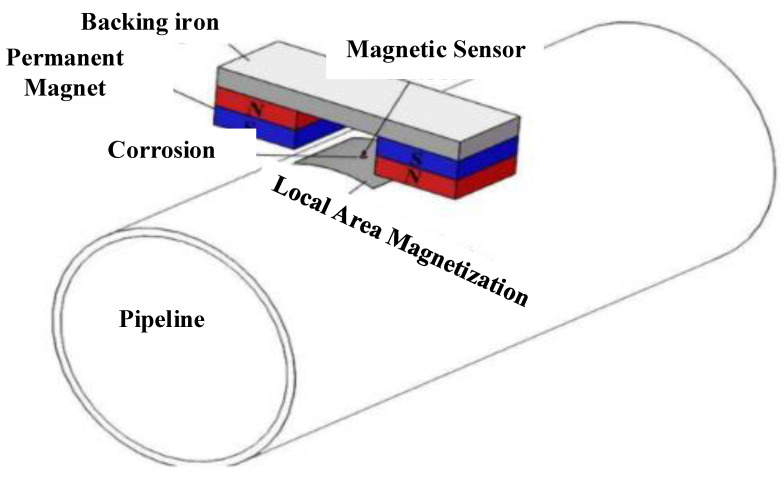
MFL framework for corrosion detection and monitoring [72].

Magnetic particle inspection (MPI) is a subset of MFL. It determines the amount of magnetic flux that is lost due to corrosion [71]. In practice, a permanent magnet with direct current (DC), AC, or pulsed stimulation provides magnetization. As shown in Figure 8 [72], the inspection system mainly consists of a sensor-generated magnetic signal, PC, tri-axis transmission system, and serial port server. Qu et al. suggested a method for estimating cable corrosion breadth using spontaneous MFL (SMFL). Three mutually perpendicular scanning paths can be provided using a three-axis transmission system, which is operated with three motors. To extract multi-dimensional MFL signals, the magnetic sensor developed by Honeywell HMR2300 has been utilized. This sensor is a massive magnetoresistance sensor with a ±2 gauss range. The resolution is estimated to be approximately 70 micro-gauss. Hence, the data extracted from the experiment can be analyzed based on signal processing tools and other quantitative assessments using PC. From another aspect, MFL is only useful for determining corrosion in ferromagnetic materials.

Pipeline inspections frequently use MFL procedures. For carbon–steel pipeline inspection, the authors in [73] introduced filtering methods, including adaptive filtering and wavelet decomposition approaches for denoising the extracted signals. To quantify corrosion, a high-resolution SMFL was developed by [74] to create magnetic fields. The SMFL is also utilized to detect leakage and mass loss of the pipeline structure and identify the location of the corrosion. The authors of [75] used MFL with sensors to investigate the feature named speed variable for corrosion detection in metal pipelines. The detection of sensitivity and penetrating depth is improved owing to the abundance of components in the speed-changing signal.

### 4.6. Microwave Monitoring Method

The range of microwave frequencies is 300 MHz to a maximum of 300 GHz. Microwave waves may easily pass through dielectric-coated materials without considerable attenuation, allowing interior structures to interact with microwave data [76]. The microwave impulses would then be reflected fully off the metal. As a result, these signals pass through corrosion and fault sites twice, increasing the likelihood of detecting them beneath the covering. The microwave ND methods measure the scale or phase in assessing the sample by monitoring sending or reflected microwave data. Moreover, lift-off and inspection frequency have an impact on reflection and transmission quality. By contrast, Figure 9 in [77] depicts the microwave ND experimental setup. A coaxial waveguide probe has been positioned on top of the specimen and down the experiment with a predetermined lift-off. Excitation signals are created by the vector network analyzer and retrieved frequency spectrum information from reflected signals. A PC controls the analyzer and provides the measurement signals to the PC over the General-Purpose Interface Bus. The X-Y scanner connects to a controller using a parallel connection. MATLAB software is utilized to build the XY scanner and analyzer that operate together during the measurement. Ultrasound waves can readily pass-through dielectric coatings.

Zoughi [78] detected steel corrosion on concrete with a thickness of up to 500 mm using a three-dimensional microwave camera. The authors in [79] explored an outline of microwave and millimeter-wave-based ND processes. Detecting corrosion and pitting precursors in aluminum and steel-backed insulated buildings was one of the many uses highlighted. The authors in [80] employed a 2.4-GHz aperture microwave patch antenna integrated with Electromagnetic Band Gap and a high resolution at approximately 14 mm depth to assess steel bar corrosion in civil constructions. The near-field and far-field microwave ND methods control the reflection coefficient in scaling and phase-shifting to identify corrosion [81,82]. The far-field mode is suitable in terms of spatial resolution, signal processing, and interpretation, including sensitivity to a relative location between specimen and antenna. Nonetheless, this mode necessitates large-aperture antennas to obtain adequate spatial resolution. Large antennas are impractical and cumbersome in most situations. Indoors, the near-field mode eliminates the effects of weather, electromagnetic interference, and other factors. Near-field microwave imaging systems using open-ended rectangular waveguides are extensively utilized in ND disciplines. The specimen picture is created by the scanning microwave synthetic aperture radar (SAR) during the examination and recorded reflected signals. Later, these signals are utilized to build two-dimensional intensity raster images. A split-ring resonator sensor with super-resolution capabilities is employed in [83] for composite imaging. The authors in [84] developed a system integration with an open-ended rectangular waveguide sensor with an operating frequency of 24 GHz to identify and classify flaws in nonceramic insulators. A one-of-a-kind artificial neural network was deployed to detect and categorize faults. Microwaves, from another aspect, have a hard time penetrating conductive materials. Therefore, they can only detect surface corrosion, leaving deeper corrosion undetectable.

### 4.7. Terahertz (THz) Monitoring Method

**Figure 10:**
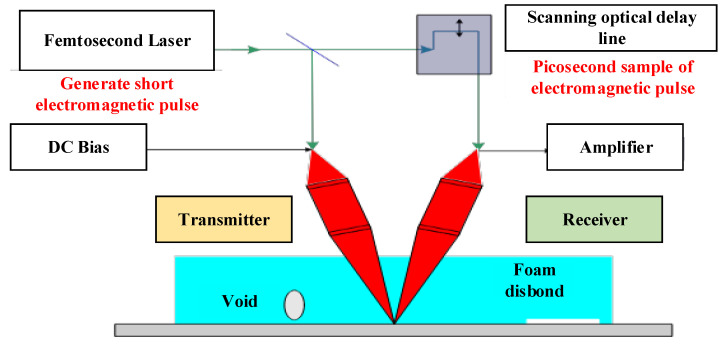
Terahertz method for fuel tank corrosion detection and monitoring under coating [10].

THz is defined as electromagnetic waves, which provide a range of frequencies from 0.1 to 10 THz. THz radiation has wavelengths ranging from 0.03 to 3 mm, which fall between the microwave and infrared spectra. The THz pulse, including known wavelength, is utilized to light the target specimen during experimentation. The emitted pulse is also analyzed in the near radiation source after interacting with the specimen during the investigation. The internal structure of the tested material is discovered by monitoring the fluctuations in the THz waves that are affected by the sample’s dielectric properties or discontinuity. Figure 10 shows that a typical THz monitoring system investigates to detect faults under the coating materials of shuttle fuel tanks in the space shuttle [10].

THz waves penetrate mostly non-metallic dry materials including coating, foams, glass, ceramics, and composite materials [85]. Thus, THz technologies are mostly utilized for ND applications, which can be separated into two categories, namely, pulse THz and continuous THz. The THz-based ND method provides specific benefits for the detection of the internal fault of the nonmetallic materials compared with other ND monitoring methods. The THz wave can also penetrate opaque materials and find defects inside them.

Insulating materials have also been inspected using THz-based ND testing. The capability of THz-based imaging has been investigated for corrosion detection undercoating by the US Army Research Laboratory and NASA. THz imaging can detect corrosion beneath the coating, which causes supposedly flat surfaces to become harsh and uneven [86]. The THz wave, from another aspect, is unable to permeate metallic materials, which restricts its use. High pricing and extensive water absorption are major drawbacks of THz-based systems [87].

### 4.8. Thermography Monitoring Method

**Figure 11:**
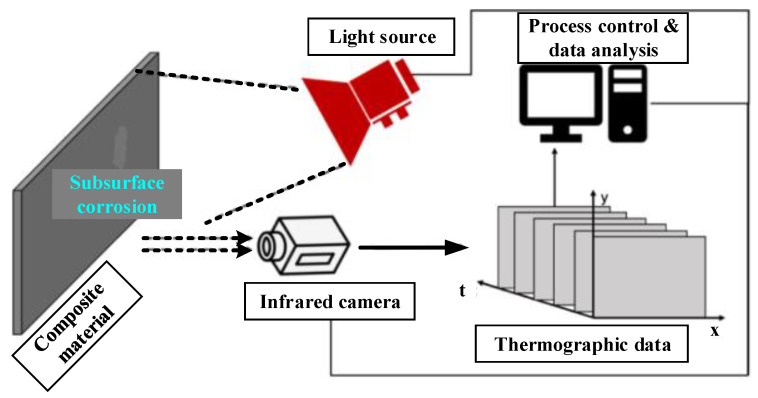
Thermography setup for corrosion detection and monitoring [88].

Thermal variance for a specified sample that responds to a stimulus is measured using thermography monitoring. More advanced kinds of thermography have resulted from advancements in IR cameras. The benefits of thermography are that it can be performed in real-time and non-contact, and it can inspect a large area in a short amount of time. Infrared light is undetectable to the naked eye, is released by the objects owing to their thermal environment, and is used to create a thermal image with an IR camera, as shown in Figure 11. The detection of infrared thermography is based on temperature differences [88]. Thermography is divided into two types: active and passive [89]. The use of a stimulus that provides heat to the target to determine a range of its characteristics is known as “active thermography” (AT). Defects or corrosion can exist among the features obtained. The term “passive thermography” refers to the measurement of temperature variations between the target material and surrounding materials, including the ambient temperature. AT-based approaches are often the most widely employed. Infrared thermography was used by Sfarra et al. [90] to find cellular features in honeycomb architectures. Infrared thermography has been investigated for pipeline corrosion detection undercoating [91]. The high cost of quality thermal cameras is one of the disadvantages of the IR approach, but recent advancements have dramatically reduced this cost.

### 4.9. Radiograph Monitoring Method

**Figure 12:**
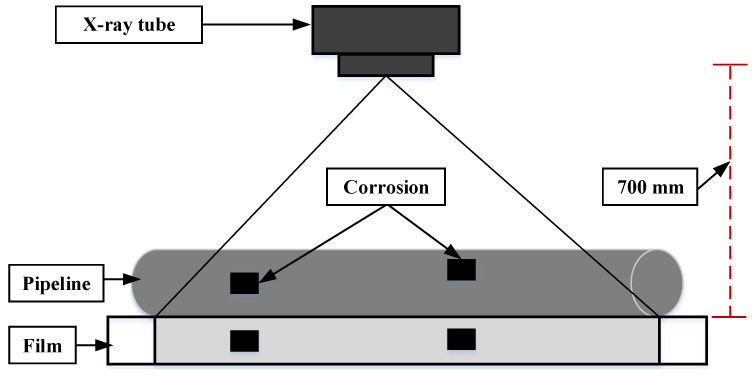
Schematic framework of radiography setup for corrosion detection and monitoring [93].

ND radiography monitoring is becoming one of the most common systems, which change the attenuation of penetrating radiation in structures as a function of radiation energy, thickness, and density. The corrosion causes the variation of the transmitted radiation intensity. As a result, radiographs are effective in detecting flaws, corrosion, and welds in metal [92]. An X-ray tube integrated with a 160-kV generator and a (1:5 mm × 1:5 mm) focal spot was used to investigate corrosion in coated steel materials, as shown in Figure 12 [93]. The current and voltage of the X-ray tube are tuned to 10.7 mA and 150 kV, respectively. The exposure period is set to 190 s for mapping corroded regions in 11-mm thick tested samples. To get the concealed corrosion during the radiography experimentation, the single wall and picture approaches are applied. With the sample under test, the film is kept intact. The gap between the film and the X-ray source was set up at 700 mm to reduce the distortion and geometric un-sharpness. The suitable image quality indication is used to produce a radiographic image sensitivity of 2%. The radiograph image was digitized with a 50-micron resolution using the Model Array 2905 film digitizer.

Digital, Profile, flash, and real-time radiographies are some more radiographic techniques developed by the researchers [94]. These methods rely on the utilization of gamma or X-rays to photograph a structure’s profile or to deliver information about the structure’s internal thickness. Discontinuities in insulated metals can often be observed easily. Despite its high cost and the fact that ionizing radiation causes health and safety dangers, radiography is nonetheless routinely used [95]. Digital radiography has improved to decrease the need for film, which has resulted in cost savings. Apart from the aforementioned concerns, however, radiography monitoring methods have some major limitations. Such methods are neither suited for detecting surface corrosion, for example, nor possible to obtain quantitative information for estimating the depth of the corrosion.

### 4.10. Acoustic Emission Monitoring Method

**Figure 13:**
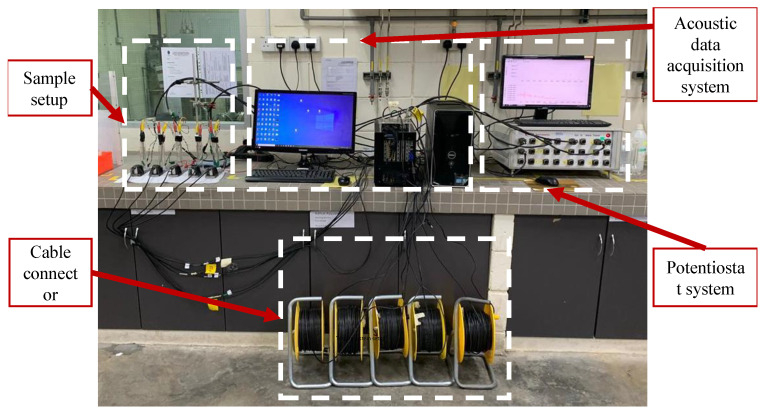
The LPR test setup for corrosion detection and monitoring using acoustic emission [96].

A transient elastic wave is created when stress energy is released rapidly on the surface of the structures. As a result, AE signals can monitor the dynamic process to detect degradation of the materials owing to corrosion. When the external force (pressure and load) and other environmental factors (pH, $CO_{2}$, temperature, and others) are utilized on the structures, the energy is released. Considering these forces and factors, the stress wave creates travels to the surface of the materials where AE sensors can measure them for further investigation. Figure 13 shows the LPR experimental setup for carbon–steel corrosion monitoring using AE.

Carbon–steel pipeline condition monitoring can be used to track the pipeline’s structural integrity or the process’s status (process condition monitoring, process monitoring). As a result, a monitoring system must have the ability to detect structural and process faults (e.g., fractures or leaks; undesirable fluid characteristics, for example, erosion or cavitation). When material qualities are established, AE evaluation can be used as a foundation for ongoing plant monitoring, improving structural safety, and reducing inspection costs. For example, Wood and Harris [97] proposed that 20-year data on large cryogenic storage tanks can be used to estimate remaining life and grade structural integrity. Moreover, AE methods have been employed to identify uniform or pitting corrosion in the majority of investigations. Zhang et al. [98] investigated the use of an AE waveform recorded by the sensors to detect the stress corrosion of the steel material. The AE approach was utilized by Wu and Byeon [99] to assess the course of pitting corrosion for the steel structure. The researchers confirmed that AE may be used in basic research for the assessments of pitting corrosion because it has several advantages compared with other methods. The authors in [100] proved the utility of AE for the early identification of corrosion in concrete buildings.

**Table 3:** A summary of ND corrosion monitoring techniques.

ND Technologies	Pros	Cons
Ultrasonic [54]	Long-range and automated monitoring capabilities; deeply accessible in materials; fast corrosion monitoring in both internal and external surfaces	Required accessible and smooth surface; coupling materials and reference standards are also needed.
Laser ultrasonic [56]	Offered high energy and non-contact broadband alternative; Produced corrosion images by scanning the tested sample	Utilities of this method is limited and it is sensitive to surface breaking issue.
OFS [60]	Long-range and continuous monitoring capabilities	The distributed sensors may isolate from the structure and difficult to detect corrosion location in the pipeline.
Eddy current [64]	It is fast and most commonly used in conductive materials; portable and cheap	It is sensitive to skin effect and surface oriented.
MFL [72]	It cost-effective and portable; deeply penetrate the materials and an effective for corrosion localization	it is sensitive to the material’s surface and limited to ferromagnetic materials.
Microwave [77]	It is powerful in terms of energy and easily accessible to the coated materials;	It has difficulties in penetrating conductive materials; limited with surface corrosion detection whereas the deeper corrosion was undetectable.
THz [10]	It is highly sensible; better resolution; easily accessible in coated materials	It is sensitive to the environment; costly and complex wave interaction.
Thermography [88]	It has long-range capability; fast remote sensing and high sensitivity	Required heating and cooling processes; not suitable for thick materials and costly.
Radiography [93]	It is suitable for coated and thick materials; cost-effective and real-time	it is not appropriate for surface corrosion detection and provides only qualitative information.
Acoustic emission [96]	It is suitable for ultra long range and long-term corrosion monitoring for coated materials; non-intrusive and real-time; Highly sensitive; No energy require to be supplied (unlike ultrasonic)	It is sensitive to environmental noise, high sampling rate and requires advanced signal processing tools.

Based on the above discussion on several corrosion monitoring methods, the impact of AE method is better compared to others in terms of corrosion growth monitoring, localization, cost of long-term monitoring set-up and large-scale monitoring. The AE approach is a highly sensitive passive test method over other ND methods which is used to detect the micro-changes and depth of the corrosion in a monitoring structure. Moreover, AE method detectability is another important parameter which can be local, semi-global and global in terms of the detection range [33]. The global AE approach detection range may vary from 2 m to 120 m or even more distance in between AE sensors [101]. This detection distance mainly depends on some other factors such as temperature, velocity, environmental and pipeline conditions [101]. Similarly, the OFS based ND method can also be both local and global in terms of the detection range [102]. Traditional OFS can only detect local or point measurements but SMARTape distributed OFS detection range can be kilometric (Up to 300 km) [102]. However, the distributed OFS approach is practically complex and expensive in implementation than the AE method. On the other hand, majority of other ND methods (ultrasonic, laser ultrasonic, eddy current, MFL, microwave and so on) are local detection methods, where they are only able to measure on the sensor point [44].

AE techniques can be utilized for qualitative and quantitative analysis. Further ND procedures are essential to achieve quantitative data in terms of the extent and corrosion depth estimation. However, environmental noise has a significant impact on the recorded AE signals. Signal discrimination and noise reduction techniques are therefore critical in real-world applications. Considering the limitations of recorded AE signals, advanced signal processing and analyzing methods are introduced to improve the AE signals and to facilitate pipeline corrosion monitoring and assessments [50,103]. The next sections of this study will emphasize and explain the importance of AE methods and AE signal processing techniques.

## 5. Importance of AE Techniques for Corrosion Monitoring

The AE method permits us to extend our hearing to detect sounds of higher frequencies and lower intensities. Damage initiation, microcrack propagation, fracture of reinforcement particles, corrosion growth slip, phase changes, stress or load identification, fault slip, and dislocation movements in steels, metals, composites, wood concrete, rocks, and geologic materials are all examples of processes we can now listen to [104,105,106]. Typically, AE maintains the sound frequencies up into the high range of ultrasonic. The AE measurement is normally recorded in the range between 30 kHz and 2 MHz [107]. The operating frequency during the LPR test is in the range between 5 and 20 kHz [96]. Moreover, the detection of AE activity concentrates on the frequency range between 30 and 150 kHz during the hydrogen evolution process [50]. At higher frequencies, the AE is not enough intense in most cases, and the material absorbs large parts of the signal. In general, at lower frequencies, background noise disturbs the measurement, for example, vibrations from vehicles and noise from pumps or flowing mediums. As for carbon steel, the AE technique has been used extensively because of the complexity of the corrosion development within this material. The AE method can be distinguished from the other monitoring techniques utilized to analyze corrosion growth, localization, and failure process, for example, optical microscopy, which utilizes the information recorded by the material damage process while and where they occur. The AE signal depends to a great extent on the type of corrosion detection system and the type of structure being investigated. The AE technique has the following unique features [108]: (i) AE is non-directional; the emitting sources radiate the energy in every direction. (ii) AE is sensitive to defect growth and changes in the material rather than to the static presence of defects. (iii) the AE method has the ability to locate damage that acts as an emission source while it occurs. (iv) AE is a passive technology, in the sense that no external energy needs to be supplied, but energy arising from the defect within a structure itself is utilized. (v) Although AE is primarily used as a local method to monitor a certain location of a structure, with the increased number of sensors, it can be used as a semi-global or global technique to monitor a larger area or a complete structure.

## 6. AE Data Processing for Corrosion Detection and Classification in Carbon–Steel Pipeline

**Figure 14:**
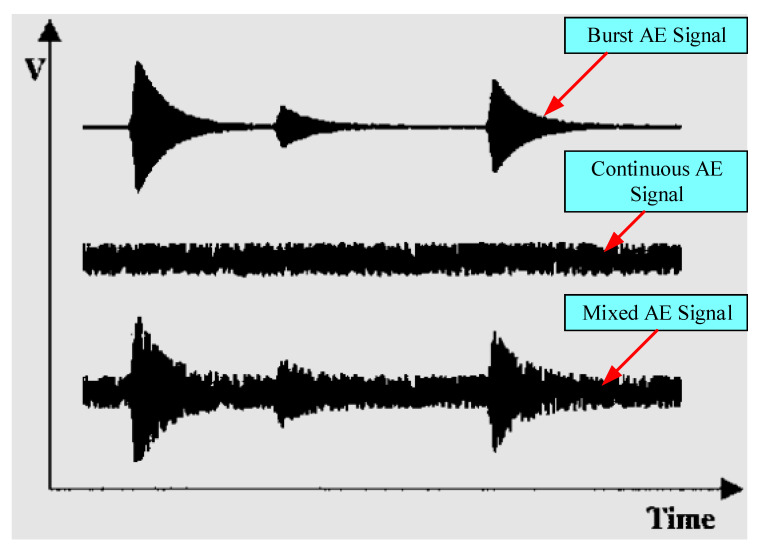
Different types of AE signals recorded from the experiments [109].

Burst, continuous, and mixed AE signals may all be recorded during experimentation, as shown in Figure 14. Burst signals record the shape of discrete transients. An exponentially decaying sinusoid is often used to imitate the shape of a burst waveform. A continuous AE signal has a random oscillatory appearance and is caused by several burst-type overlapping AE signals with indistinguishable magnitude. In most real-world environments, burst and continuous signals are extracted because both can be important signals for analysis or one of them can be noise.

Most of the AE time-series data contain frequency information ranging from 0.1 to 1 MHz. The temporal and/or frequency domains can thus be used to select the features of those signals. Although time-domain signal analysis is commonly employed in the applications of AE signals, AE signal is comparatively simple [50]. The frequency analysis of the AE signal can often present an indication of the source type. The spectral decomposition method can be utilized to distinguish several propagation modes [50,110]. Moreover, the fast Fourier transform (FFT) frequency-domain transformation method is employed to determine the type of defects in valves and each defect represents a unique spectral signature [111].

The authors in [112] introduced a method to calculate the average diameter the length of glass particles based on AE signal spectral peaks when the cylinders were tumbling. Owing to the dispersive media, reflections, sensors, and multiple routes, the wave spectrum sends at the variation of speeds and wave guiding qualities in complex materials, which may produce distorted signals. Spall et al. [113] investigated and found the difficulty in recognizing signals received by transducers far from the source. The author in [114] reduced the strength of a Bruel Kjaer type 2637 preamplifier by the factor of 10 (20 dB) to avoid signal distortion recorded from pencil lead breaks experiment on a cutting tool. Moreover, the AE signal should be filtered as early as feasible in the processing chain, right after the inevitable buffering as suggested in the proposed work.

Mathematical transforms are commonly used to extract essential information from raw signals that are not immediately available from their time evolutions. The Fourier transform is utilized to split the AE signal into its frequency information [115], which is the most used tool in AE signal processing. The FFT is a mathematical procedure that decomposes numerically discrete time-domain signal data into the frequency-domain in a short amount of time while avoiding round-off errors [116]. Another approach is power spectral density (PSD) estimation, which uses the FFT to present how the energy of the signal is distributed in the frequency-domain representation [115]. The Welch approach in [117] employed to estimate PSD using MATLAB software. The Welch method computes PSD by the division of time-domain signal into segments (sometimes overlapping) and utilizing the FFT to assemble each segment of a changed periodogram. Then, these periodograms are averaged to reduce the variance of the PSD estimation.

**Figure 15:**
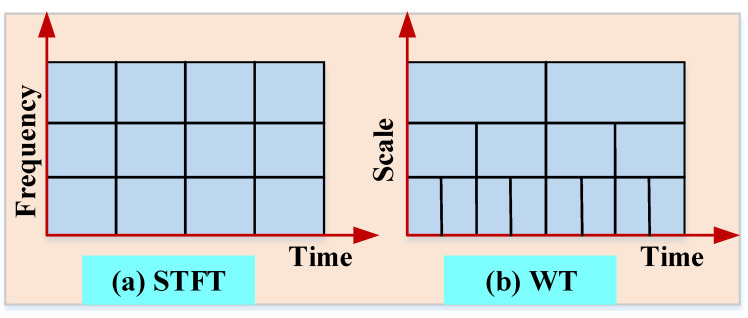
Resolution of AE signal after transforming based on (**a**) STFT and (**b**) WT [77].

The FFT and PSD both decomposition methods need signals to be stationary, including the frequency power remaining consistent throughout time [118]. A range of time-frequency analyzing tools, for example, wavelet transform (WT), and Short-Time Fourier transform (STFT) are widely available for non-stationary signals. The STFT is a basic method that uses a time-varying spectrum to explain the signal’s energy distribution. By analyzing the raw signal with the same window size, the STFT can offer a consistent resolution for the frequencies of the signals. Nevertheless, a trade-off exists between the resolution of the temporal and frequency, as shown in Figure 15a. Moreover, the WT performs a multi-scaling analysis on the signal, in which the time resolution and frequency resolution vary in the plane of the time-frequency, as shown in Figure 15b, with a longer time interval. The WT provides more precise information about the low-frequency and shorter intervals of time and provides more precise information about high frequency, thereby enhancing the overall representation of the time-frequency attributes.

Noise from the environment, for example, is common in AE signals and must be eliminated to facilitate signal interpretation. To define the AE signal components and reduce noise, time-frequency analysis might be utilized. For example, Ng and Qi [119] proposed a wavelet-based AE energy technique in which the ratio of the energy of the reconstructed signal has been computed to detect minor information in the AE signal without compromising the original signal integrity. The authors employed a wavelet-based decomposition method to extract AE energy with fatigue metrics. In addition, the authors in [120] introduced the wavelet decomposition method integrated with the Gabor wavelet, which is considered a useful tool for studying wave propagation processes in structures. The information retrieved using the WT decomposition method can be used to precisely evaluate the dispersion behavior in velocity. In general, the WT is an effective way to analyze AE signals as it gives frequency and temporal information.

## 7. Corrosion Prediction Models and Reliability Assessment of Carbon–Steel Pipeline

**Figure 16:**
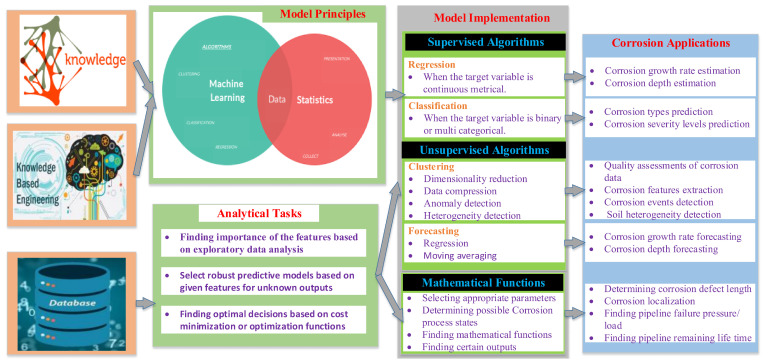
Diagram of data-driven corrosion modelling and its applications.

For asset managers, the dependability of carbon–steel pipes is critical. Methods of reliability analysis are often used to predict how long structures will last and, in turn, to make sure they are safe. In the development of novel corrosion prediction models, the calibration has been performed initially utilizing the data that have been collected from sensors in Section 3. The accuracy of the corrosion prediction models is a crucial factor in the accuracy achieved by the reliability prediction models. Accordingly, the maintenance works have been scheduled and performed effectively [121]. Predictive models for carbon–steel pipeline corrosion can be classified as probabilistic, deterministic, statistical, fuzzy logic, and knowledge-based models. Figure 16 depicts a diagram of these data-driven models and their applications in the field of pipeline corrosion. The methods used to estimate the corrosion of the carbon–steel pipeline are discussed in this section. In addition, the reliability of corroding carbon–steel pipes is investigated.

### 7.1. Deterministic Model

Examining the relationship among multiple important parameters excluding the unimportant parameters, the deterministic models have been carried out based on laboratory experiments, including the corrosion data that are collected from the real environment. Numerous deterministic models have been introduced recently in [122,123,124,125] to model corrosion assessments in carbon–steel pipelines in the OG industry. Non-experts may easily build up and comprehend these models. Deterministic models, from another aspect, ignore the randomness of the corrosion process and hence fail to reflect the true character of corrosion. Furthermore, the results of these models are not suitable for the investigation of the physics of corrosion [126].

### 7.2. Statistical Model

Based on the corrosion and failure data collected from the experiments, statistical models have been developed to assess and predict the future state of the pipeline structure. Several statistical models exist in the literature, such as linear regression, nonlinear regression, Bayesian inference, and Markov chain models for carbon–steel pipeline corrosion prediction [127,128,129,130,131,132]. Expert opinions have been used to calibrate the distribution of the related parameters. Romanova et al. [133] used data from pipe segments to make an evolutionary polynomial regression model, which can predict how fast pipe segments will corrode and how long they will still work. A statistical analysis, including joint multivariate normal distribution fitting, has been performed by Del Giudice et al. [134]. The amount of the input dataset and the quality of the data have a big impact on the statistical models’ accuracy. Most engineering problems do not have easy access to high-quality and large enough input data, which makes statistical methods less useful.

### 7.3. Knowledge-Based Model

Knowledge-based models encompass several artificial intelligence (AI) models, including ML, for forecasting the future corrosion states in the pipelines. Knowledge-based approaches use various statistical analytic methods to process the input data to predict patterns and effects [96,135,136,137,138]. In other words, to predict the desired output, a machine-learning algorithm learns from input data and updates automatically by the variation of the model parameters. The learning process has three types: unsupervised, supervised, and reinforcement learning. The algorithm in unsupervised learning, from another aspect, learns from unlabeled data to disclose latent patterns in the input data. A combination of labeled and unlabeled data can be used in a semi-supervised learning algorithm to improve the effectiveness of the learning process. The data are labeled, and the outcome is predicted in supervised learning. Moreover, reinforcement learning requires making the best possible decisions in an unpredictable environment to maximize the reward through trial and error. When input data are abundant, machine-learning models can outperform the other stated methods in terms of accuracy and effectiveness. These models are robust in the presence of noisy or missing data because they integrate linear and non-linear linkages between key factors.

### 7.4. Probabilistic Model

The corrosion of a structure cannot accurately predict the absence of data along with statistical, deterministic, and knowledge-based models. This problem can be solved using probabilistic models [139,140,141]. The unpredictability of the deterioration is measured by the probabilistic models’ process, and thus, it detects accurate mimic stochastic corrosion processes. Implementing these models, from another aspect, demands a deep understanding of mathematics and the physics behind the corrosion processes. Non-experts may find it difficult to use these tactics because of the usage of relatively advanced probabilistic approaches in generating the models. The pipeline corrosion process mainly depends on time. Owing to the nature of corrosion, long-term studies should be carried out on corroded structures for reliability analysis. Among the time-dependent reliability analysis methods [142], the Gamma process concept [143] and time-discretized Monte-Carlo Simulation (MCS) [144] are mostly utilized for the reliability prediction of corroded materials. Assuming that no correlation exists between the failure modes, the usability and failure modes are arranged in series and parallel designs. The authors in [142] introduced a reliability prediction analysis, which is multi-failure and time-dependent. The proposed model predicted the service life of the corroding carbon–steel pipeline. If the correlation between different time steps is ignored, then MCS can only capture the degrading time-dependency [144]. This problem was resolved by using the Gamma process idea in [145]. For modeling gradual degradation, the monotonic falling pattern of the corrosion is assessed by the Gamma process. The authors in [146] employed the Gamma procedure to test the reliability of corrosion-affected carbon–steel pipes. To mimic how corrosion varies over time, the corrosion model based on power law was employed as the form function of the Gamma distribution.

### 7.5. Fuzzy Logic Model

Fuzzy logic relies on expert opinion and specialized expertise to predict pipe deterioration. These models can be beneficial when there is a shortage of data or when the available data are hazy and confusing. In fuzzy logic, three processes exist: data fuzzification, fuzzy inference, and data defuzzification [147]. In the fuzzification process, the input data are transformed into a linguistic format that can be understood by humans. “Fuzzy inference” is the process of employing fuzzy logic to map from the input to the output. Defuzzification is the mechanism of creating measurable results in a fuzzy logic framework. For defining the rule set and determining the defuzzification process, the application of fuzzy logic-based models needs a high level of expertise. In particular, fuzzy logic models in the corrosion evaluation of carbon–steel pipes can be found in the works of [148,149,150,151].

It may be concluded that the right corrosion estimation model should be chosen based on data availability, skill level, necessary prediction accuracy, and computational power. If no data on the pipeline network’s corrosion status are available, or if the available information is vague and ambiguous, then fuzzy logic models can help by relying on expert opinions and technical judgment. Probabilistic models are useful possibilities for predicting the future condition of deterioration in carbon–steel pipes when data are scarce. Considering the probabilistic character of these models, they can be used in a reliability study to forecast the likelihood of corroding pipes failing. To achieve this goal, we need considerable computing power and experience in probabilistic modeling and structural analysis. In the presence of a large amount of data, statistical and knowledge-based models outperform probabilistic models in terms of accuracy, computational cost, and necessary skill. Using knowledge-based models, a high level of automation in the dependability assessment of corroding pipes can be achieved, owing to recent improvements in the Internet of things, development of the sensors, handling techniques on big data, and the high processing capacity of the computers today.

## 8. Discussion with Future Perspective

Corrosion is still a major challenge for a variety of industries ranging from OG to sewer water. The future perspective appears to be primarily focused on creating various systems for corrosion detection, categorization, reliability prediction, and prevention. For detecting corrosion and safeguarding pipelines from corrosion, a variety of innovative active and passive sensors have been proposed. These types of sensors can be utilized to predict the reliability of OG pipelines by using various corrosion models. Recently, corrosion assessment, characterization, long-term monitoring, and reliability prediction analysis methodologies are included: the real-time data obtained by the data acquisition system, multivariate control systems, smart remote sensing components, wireless communication, and networking. Corrosion detection of the surface or subsurface, severity classification, the quantification of corrosion dimension, and advanced data pre-treatment techniques were used to clean the corrosion data, resulting in significant gains in the identification of the size and depth. However, more complicated signal processing approaches are necessary to enhance the ND system’s sensitivity and quantification ability.

Furthermore, recent studies looked at the possibility of ensemble learning, such as bagging, boosting, and modified bagging, in the OG pipelines corrosion prediction using data mining modeling. Asset managers can avoid pipeline network failure before the system fails by adding diverse corrosion modeling methodologies into reliability analysis methods, such as deterministic, statistical, knowledge-based, probabilistic, and fuzzy logic models. This method saves millions of dollars and makes sure that the structures and the people who use them are safe.

The variation in conductivity, permeability, and permittivity, including changes in thickness, are all elements that contribute to corrosion. These factors have an impact on the likelihood of corrosion detection. Owing to the intricate interaction of various elements, no universally applicable corrosion detection method exists. As coating layers make a big gap between sensors and the surface being checked, none of the above ND methods can handle all the problems that come up when trying to find corrosion under the coating. More than just the detecting capabilities must be considered when choosing a ND technique. These factors include application, equipment mobility, inspection area, inspection schedule, types of materials, accessibility, expenses, and projected corrosion types. When the coating is used, certain procedures offer quantitative information but perform badly. As a result, new corrosion monitoring systems are needed for long-term monitoring operations with low costs and risks.

This study begins with a review of the literature, followed by the analysis of corrosion detection methods employing acoustic, ultrasonic, radiographic, electromagnetic, and thermographic approaches. Owing to the thick coating layer, most approaches are constrained with regard to online in-situ corrosion monitoring undercoating. Engineers frequently employ clumsy, costly equipment to inspect holes in the layer of coating to transmit signals over the distance of an insulated structure to solve this problem. To solve the issues of corrosion undercoating, new ideas and methodologies, signal processing tools, the coupling of SHM and ND, intelligent inspection systems, and the installation of unmanned techniques are all concerns that need to be investigated.

The integration of AI in an intelligent inspection system can help a corrosion detection system work more efficiently. Distinct types of corrosion necessitate different treatments because different types of corrosion might occur during material testing. Consider uniform corrosion, which occurs when all surface area corrodes simultaneously (or nearly the same). Uniform (or broad) corrosion is reasonably easy to track and forecast. This sort of corrosion reduces the thickness and weight of the metal, which is the most common type of corrosion that needs to be identified to improve the coating’s manufacturing quality. As a result, an intelligent inspection system is required to classify the corrosion kind. AI approaches can be utilized to increase uniformity and save inspection time. Corrosion has been detected using neural networks, normal computer vision methods, and deep learning methodologies. Corrosion can be detected horizontally and vertically by autonomous robots. The ND system’s safety and efficiency can be greatly enhanced. To allow autonomous inspection capability, however, lightweight equipment and powerful detecting systems are required.

## 9. Conclusions

Advances in ND corrosion methods and assessment of corrosion undercoating in carbon–steel pipelines led to the development of various sensors and monitoring methods in searching for improvement in the OG industry. The AE monitoring method portrays promising results due to its capability in real-time corrosion monitoring of pipelines subjected to certain challenges. The challenges have been highlighted with several recommendations for solutions. Successful implementation of real-time corrosion monitoring in pipelines using AE is a step forward toward the emergence of smart pipeline monitoring to keep up with the future trend through the development of corrosion detection, classification, reliability prediction and prevention techniques. In conclusion, the realization of continuous real-time pipeline corrosion monitoring technology is possible considering the existing shortcomings are taken into account and resolved systematically for a better pipeline system. This can help the OG industry in predicting future outcomes more accurately and plan for unknown events to avoid consequences of failure that can be catastrophic due to the potential hazard to the Human Health and Safety Loss.

## Figures and Tables

**Figure 9:**
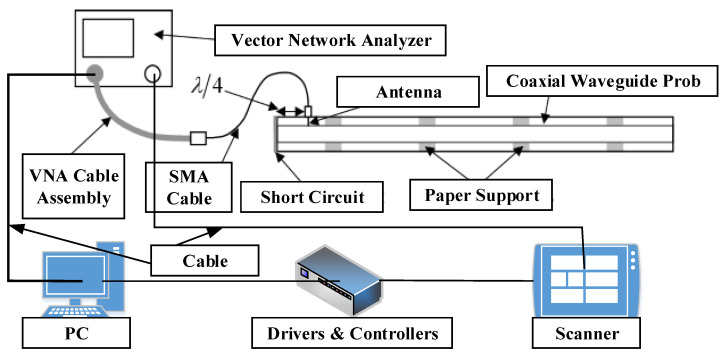
Microwave framework for corrosion detection and monitoring under coating [77].

**Table 1:** A summary of several kinds of corrosion in a carbon–steel pipeline, its causes and prevention methods.

Corrosion Types	Causes	Preventive Methods
Uniform corrosion	Open atmospheres, soils, $H_{2}O$, pH etc.	Coating and chemical treatment.
Pitting corrosion	chemical and mechanical attacks	Oxide film and pH management.
Crevice corrosion	Metal-ion cells and oxygen cells	Welding and non-absorbent gaskets.
Galvanic corrosion	Electrochemical potential	Zinc coating.
Cavitation corrosion	Variation of rapid pressure	Minimizing bubble formation and collapse.
Erosive corrosion	High velocity	Controlling velocity and utilizing copper-nickel alloys.
Stray current corrosion	DC flowing near the soil and discharge	Coating and cathodic protections.
Stress corrosion cracking	High stress, loading and temperature	Threshold management and monitoring.
Microbiologically induced corrosion	Anaerobic and aerobic bacteria as well as other enzymes	Chemical treatment and inhibitors.

**Table 2:** A summary of different corrosion sensors and their characteristics.

Corrosion Sensors	Features	Pros	Cons
Corrosion coupon	Measure materials weight loss	Quick response, simple and flexible	Not real-time and limited with general corrosion.
ER probe	Mesure the metal loss	Remote monitoring, Environments friendly and real-time	Not suitable for liquid metal and conductive salts environments.
Electrochemical	Detect small quantity of corrosion and measure overall weight loss	Utilizing in both dry and wet gas environments	Not appropriate for conductive liquids.
Ultrasonic	Measure the wall thickness of the materials	ND, real-time, suitable for both internal and external corrosion	Not appropriate for small thin materials.
Multi-frequency electromagnetic	Detect the wall properties and measure the wall thickness	Non-intrusive, real-time and cost-effective	Limited with surface and sub-surface inspections.
OFS	Measure corrosion as a function of specimen roughness and color	ND, real-time, low cost and effective undercoating and painting	Require to measure coating and paint thickness of the specimens.
RFID	Measure the wall thickness based on resonance frequency features	Passive, wireless, low-cost and multiple resonances	Not efficient in reliability and sensitivity.
AE	Measure corrosion rate and detect microscopic damage	Passive, non-intrusive, low-cost, real-time and remote monitoring	Sensitive to background noise.

## Data Availability

Not applicable.
